# Differential Defecation of Solid and Liquid Phases in Horses—A Descriptive Survey

**DOI:** 10.3390/ani10010076

**Published:** 2020-01-01

**Authors:** Katrin M. Lindroth, Astrid Johansen, Viveca Båverud, Johan Dicksved, Jan Erik Lindberg, Cecilia E. Müller

**Affiliations:** 1 Department of Animal Nutrition and Management, Swedish University of Agricultural Sciences, P.O. Box 7024, 750 07 Uppsala, Sweden; Johan.Dicksved@slu.se (J.D.); jan.erik.lindberg@slu.se (J.E.L.); cecilia.muller@slu.se (C.E.M.); 2 NIBIO, Norwegian Institute of Bioeconomy Research, P.O. Box 115, 1431 Ås, Norway; astrid.johansen@nlr.no; 3 National Veterinary Institute, 751 89 Uppsala, Sweden; viveca.baverud@sva.se

**Keywords:** colic, equine, free faecal liquid, faecal water syndrome, feed changes, nutrition

## Abstract

**Simple Summary:**

Free faecal liquid is a condition in horses where faeces are voided in one solid and one liquid phase. The presence of free faecal liquid may cause management problems in equine husbandry and is potentially contributing to impaired equine welfare. Causes of free faecal liquid are not known, but nutritional factors such as the feeding of specific forages have been suggested to be of importance. Characterization of horses showing free faecal liquid and their feeding and management was, therefore, performed via an internet-based survey in order to map the condition further. Results showed that horses with free faecal liquid included a large variety of different breeds, ages, disciplines, coat colours, housing systems and feeding strategies, meaning that almost any type of horse could be affected. Horses that were reported to show free faecal liquid did so with all types of feeding strategies, but changes from wrapped forage to hay, to pasture, or to another batch of wrapped forage often resulted in diminished signs of free faecal liquid. Horses were also reported to have a comparably high incidence of colic in relation to published data for other horse populations. The results indicated that more detailed studies are required for a further understanding of the underlying cause of free faecal liquid.

**Abstract:**

Free faecal liquid (FFL) is a condition in horses where faeces are voided in one solid and one liquid phase. The liquid phase contaminates the tail, hindlegs and area around the anus of the horse, resulting in management problems and potentially contributing to impaired equine welfare. The underlying causes are not known, but anecdotal suggestions include feeding wrapped forages or other feed- or management-related factors. Individual horse factors may also be associated with the presence of FFL. This study, therefore, aimed to characterize horses showing FFL particularly when fed wrapped forages, and to map the management and feeding strategies of these horses. Data were retrieved by a web-based survey, including 339 horses with FFL. A large variety of different breeds, ages, disciplines, coat colours, housing systems and feeding strategies were represented among the horses in the study, meaning that any type of horse could be affected. Respondents were asked to indicate if their horse had diminished signs of FFL with different changes in forage feeding. Fifty-eight percent (*n* = 197) of the horse owners reported diminished signs of FFL in their horses when changing from wrapped forages to hay; 46 (*n* = 156) of the horse owners reported diminished signs of FFL in their horses when changing from wrapped forages to pasture; 17% (*n* = 58) reported diminished signs of FFL when changing from any type of forage batch to any other forage. This indicated that feeding strategy may be of importance, but cannot solely explain the presence of FFL. The results also showed that the horses in this study had a comparably high incidence of previous colic (23%, *n* = 78) compared to published data from other horse populations. In conclusion, the results showed that FFL may affect a large variety of horse types and that further studies should include detailed data on individual horse factors including gastrointestinal diseases as well as feeding strategies, in order to increase the chance of finding causes of FFL.

## 1. Introduction

Free faecal liquid (FFL) is a condition in horses where faeces are voided in two physical phases; one solid and one liquid phase. The solid phase can be typical equine faecal balls, or more watery and similar to cowpat faeces. The liquid phase is a brown-coloured liquid that can be voided separately or together with the solid phase. The condition has previously been referred to as free faecal water and/or free faecal water syndrome (FWS), and cases have been described in Germany [1,2,3], Denmark [4] and Italy [5], but the overall incidence of FFL is not known. Horse owners in Sweden and Norway have anecdotally reported cases of FFL in horses, and have referred to the condition as “haylage intolerance” due to an assumed association with feeding wrapped forages (including grass conserved as silage and/or haylage with dry matter concentrations from 300–840 g per kg [6,7,8]). During the latest 25 years, wrapped forages such as grass silage and haylage have partially or totally replaced hay in equine feed rations in Nordic countries [9,10,11].

Horses affected with FFL may show discomfort when voiding faeces and/or faecal liquid, such as nervous trampling with hindlegs and extensive tail swishing, but no symptoms of disease have been described [1,5]. The faecal liquid may, however, cause lesions in the skin around the anus and on the inside of the hindlegs, as well as dirty tail and hindlegs of affected horses. The causes of FFL are unknown, but feeding wrapped forages instead of hay, feeding high amounts of alfalfa, being over 20 years of age, having poor dentition and endoparasitic infections have been suggested [1]. In a German study [1], associations between the presence of FFL and intrinsic horse factors such as being a gelding, paint-coloured and low in the social hierarchy in a group of horses were found. Improvement of the condition have been reported in association to changes in the diet in a one-horse case study [5] and after faecal transplantation performed in a study including 10 horses with FFL and twelve horses assessed to be clinically healthy [4]. However, no clear associations to feed- or management-related factors [1,3,12] has been reported. Systematic collection of data on horse characteristics as well as on feeding and management of horses affected with FFL is scarce in the scientific literature. The aim of the present study was, therefore, to characterize horses showing FFL (when fed wrapped forages) and to map the feeding and management strategies of these horses. Such information is required for further studies of causes for the condition.

## 2. Materials and Methods

An online survey directed to owners and/or caretakers of horses showing FFL when fed wrapped forages was performed. The inclusion criteria were that horses should be >2 years old, showing FFL when fed wrapped forages and be located in Sweden or Norway (two countries in close proximity and with similar conditions for horse feeding and management). The inclusion criteria were given on the start page of the survey, and 3 questions in the survey were control questions ensuring that the inclusion criteria were met. The survey was created using the tool Netigate (Netigate, Stockholm) and was advertised through the website of the Department of Animal Nutrition and Management, SLU (http://www.slu.se/sv/institutioner/husdjurens-utfodring-vard/), Norsk hestesenter (http://www.nhest.no), Norwegian institute of Bioeconomy Research (http://www.nibio.no) and the website Hästsverige (http://www.hastsverige.se), a Swedish platform communicating equine research to the public. The full survey is available in Table S1 (Supplementary Materials).

### 2.1. Data Collection

The survey was open from March 2016 to March 2017 and was available in both Swedish and Norwegian language. Respondents were instructed to give information about one horse per entry, even if they owned more than one horse showing FFL. If so, it was possible for the same respondent to answer the survey several times. The respondents were asked to answer questions about the horse, how it was kept and managed, current and previous feeding and history of gastrointestinal disturbances including FFL. The survey contained 50 questions in total, divided into horse characteristics (e.g., age, sex, breed, colour, body condition score [13] and temper as judged by the respondent); training (e.g., discipline, intensity); management (e.g., type of housing system, paddock use); current feeding (type and amount of feeds), feeding and watering strategies (e.g., number of feedings, time between feedings, how feed was offered in stable and paddock, type of water source and access to salt); presence of FFL (e.g., presence and symptoms of FFL, changes in faecal appearance due to feed changes, number of affected horses kept in the same housing system); and previous history of gastrointestinal tract (GIT) diseases.

### 2.2. Data Treatment

The data on horse breeds contained over 30 different breeds but with very few individuals in several breeds. Therefore, this variable was transformed to breed type. The reported breeds were divided into 4 breed groups; warmblood type horses (Appaloosa (*n* = 3), crossbred horses of warmblood type (*n* = 64), European warmblood riding horses (*n* = 82), Lusitano (*n* = 1), Standardbreds (*n* = 15), Paint horse (*n* = 2), Pura Raza Espaniola (*n* = 11) and Quarter horse (*n* = 2)); cold-blood type horses (Ardennais (*n* = 4), Clydesdale (*n* = 1), Cold-blooded trotter (*n* = 13), crossbred horses of cold-blood type (*n* = 19), Dølehorse (*n* = 2), Friesian horse (*n* = 3), Haflinger (*n* = 3), North-Swedish draught horse (*n* = 8), Norwegian Fjord Horse (*n* = 12), Shire (*n* = 1) and Tinker (*n* = 1)); Hot-blooded horses (Angloarabian (*n* = 1), Arabian (*n* = 8), and Thoroughbreds (*n* = 2)); and native pony breeds (Connemara (*n* = 10), crossbred ponies (*n* = 25), Gotland pony (*n* = 6), Icelandic horse (*n* = 13), New Forest (*n* = 10), Shetland pony (*n* = 6), Welsh cob (*n* = 3) and Welsh pony (*n* = 8)).

### 2.3. Calculations and Statistical Analysis

Statistical analysis was performed using SAS (Statistical Analysis System Institute Inc., Cary, NC, USA) version 9.4 for Windows. For continuous variables, minimum, maximum, quartiles (Q1, Q2 (median) and Q3), mean and standard deviation was calculated. The reported bodyweight (BW) of the horse and reported feeding levels were used to calculate the daily intake of feed (g or kg) per 100 kg BW and day. Descriptive analysis was performed using PROC FREQ. During data treatment, it was found that 23% (*n* = 78) of the horses had a history of colic. The horses were, therefore, further divided into one colic and one non-colic group for comparisons of type of clinical signs during FFL episodes. Each clinical sign was compared separately between the groups using a Chi^2^-test (with expected model). Level of significance was set at *p* < 0.05.

## 3. Results

In total, 780 responses to the survey were obtained. Out of these, 12 responses represented horses younger than 2 years of age, 234 responses were for horses that did not show FFL but other types of problems when fed wrapped forages, and 195 responses were incomplete. These 441 responses were excluded from the dataset, leaving 339 full responses for further evaluation.

### 3.1. Horses and Signs of Free Faecal Liquid

**Figure 1:**
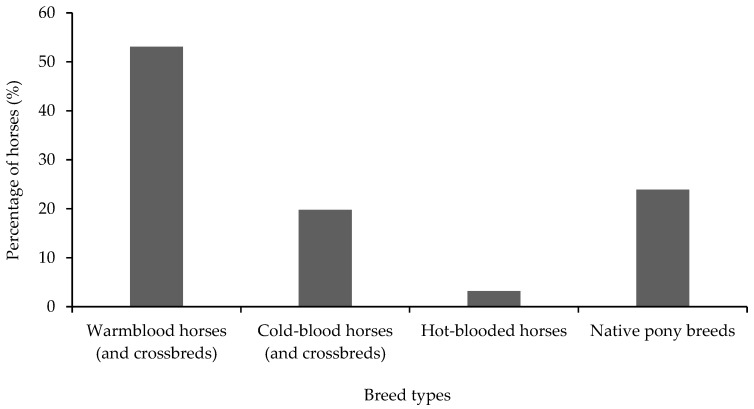
Distribution of breed types for horses showing free faecal liquid (*n* = 339).

**Figure 2:**
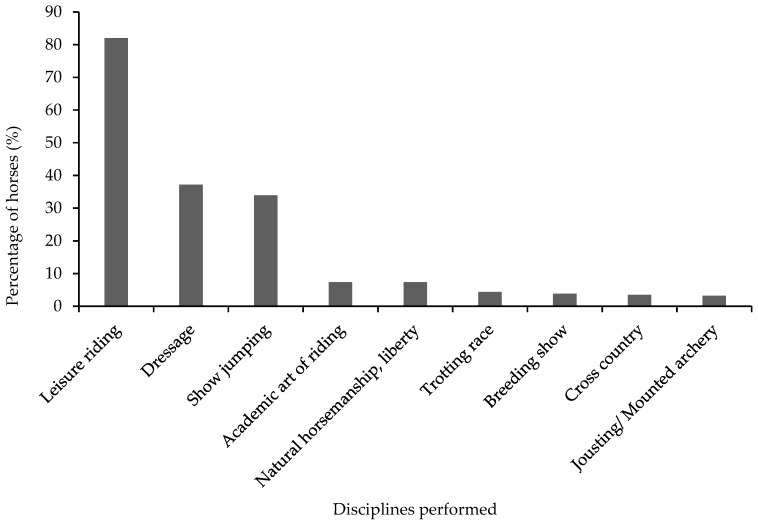
Distribution of disciplines performed by horses with free faecal liquid (*n* = 339), as reported by respondents. Multiple-choice question resulting in that the sum could exceed 100 percent.

**Figure 3:**
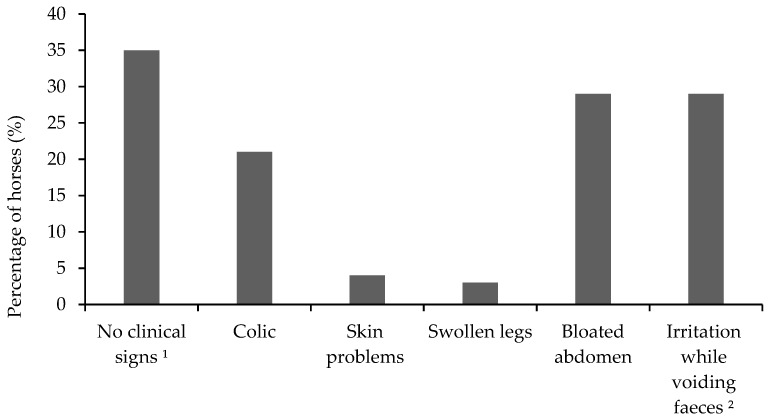
Percentage of horses showing different clinical signs associated with episodes of free faecal liquid, as reported by respondents (*n* = 339). Multiple-choice question resulting in a sum that could exceed 100%. ^1^ No clinical signs mean no signs other than free faecal liquid. ^2^ Including extensive tail swishing and/or trampling with hindlegs while voiding faeces and/or faecal liquid.

The age of the horses in the study ranged from 2.5 to 28 years (average 11 ± 5.9 years). The majority of the horses were geldings (57%, *n* = 193) (Table 1) and of warmblood breed type (53%, *n* = 180) (Figure 1). Thirty-seven percent (*n* = 123) of the horses had bay coat colour followed by chestnut (19%, *n* = 64), grey (14%, *n* = 47) and black (8%, *n* = 27). Body condition scores (BCS) ranged from 1 to 4 (on a scale of 0 to 5 [13]), with a normal distribution around BCS 3 as median (55%, *n* = 186) (Table 1). The most frequently reported disciplines horses were used for were leisure riding (82%, *n* = 278), dressage (37%, *n* = 125) and show jumping (34%, *n* = 115) (Figure 2). A majority of the horses (63%, *n* = 215) were reported to perform a low-intensity exercise (Table 1). Extended information on horse characteristics is reported in Appendix A (Table A1). Twenty-nine percent (*n* = 98) of the horses were reported to show distinct irritation manifested by extensive tail swishing and nervous trampling of hindlegs while voiding faecal liquid and/or faeces, whereas 35% (*n* = 118) did not show any signs other than FFL (Figure 3). A bloated abdomen was reported in 29% (*n* = 98) of the horses during episodes of FFL (Figure 3). Fifty-two percent (*n* = 170) of the respondents reported that only their horse in the stable showed FFL, while 48% (*n* = 159) of the respondents stated that there were more horses in the stable that showed FFL.

### 3.2. Management

The majority of the horses (79%, *n* = 271) were kept in individual boxes at night and outside in paddocks during the daytime, while 19% (*n* = 61) were kept in loose-housing systems (Table 2). The bedding materials used in stables and loose housing systems were a combination of straw and shavings (37%, *n* = 125), straw only (20%, *n* = 68) and shavings only (17%, *n* = 58) (Table 2). Horses that spent their daytime in paddocks were generally kept outside for 8–12 h per day (48%, *n* = 163), while 29% (*n* = 98) were kept outside for more than 12 h per day and 21% (*n* = 71) were kept outside for less than 4 h per day (Table 2). The type of paddocks were soil paddocks (39%, *n* = 132), grass paddocks (old grass during winter) (28%, *n* = 94) or sand/gravel paddocks (23%, *n* = 78) (Table 2). Forty-eight percent (*n* = 163) of the horses were kept on pasture for 9–12 weeks, while 21% (*n* = 71) were on pasture for ≤8 weeks and 8% (*n* = 27) were kept on pasture for >12 weeks (Table 2). The majority (55%, *n* = 186) of the horses were dewormed if faecal egg counts showed sufficiently high numbers to indicate deworming according to national guidelines (www.sva.se) (Table 2). Other deworming procedures included regular deworming more than one time per year (36%, *n* = 122), dewormed if considered necessary (7%, *n* = 25) or not dewormed (1%, *n* = 4) (Table 2). Horses were reported to have access to water by tubs, buckets or automatic waterers when kept in the stable, paddock and at pasture. At pasture, horses were also reported to have access to water by natural water sources (Table A1). Extended information on management factors is presented in Table A1 (Appendix A).

### 3.3. Feeding

**Figure 4:**
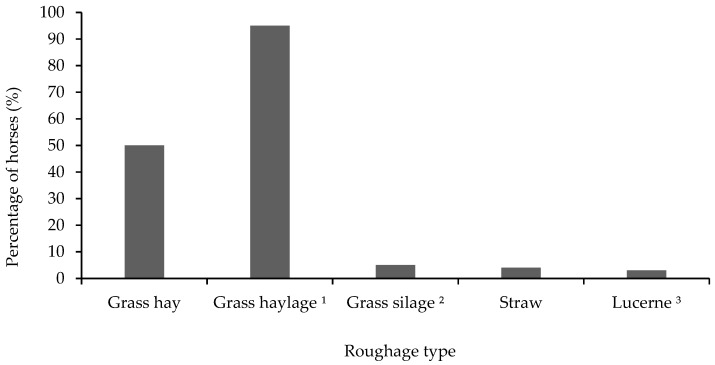
Different types of roughage fed to horses showing free faecal liquid (*n* = 339). Multiple roughages could be assigned in the survey, resulting in a sum of percentages exceeding 100. ^1^ Wrapped forage with ≥50% DM. ^2^ Wrapped forage with <50% DM. ^3^ Includes both pelleted lucerne and lucerne chaff in a dried format.

The majority (74%, *n* = 250) of horses were fed forage in meals, while 26% (*n* = 89) were fed forages ad libitum. Grass haylage (defined as in Table S1, Supplementary Materials) was offered to 95% (*n* = 322) of the horses, whereas 5% (*n* = 17) of the horses were fed grass silage (Figure 4). Hay was fed to 50% (*n* = 170) of the horses (Figure 4). In general, horses were fed roughage-dominated feed rations with on average 90% roughage, and 7% concentrates in the daily feed ration (Table 3). Daily amounts of different feedstuffs are reported in Table 3. Most of the horses (67%, *n* = 227) fed forage in meals were fed forage 3 to 4 times daily, and the time between two forage feedings seldom exceeded 8 h (Table A1). Horses that were fed in their paddocks were served forage in tubs or haynets (60%, *n* = 204), or on the ground (45%, *n* = 153) (Table A1). Eight percent (*n* = 27) were not fed forage in their paddocks (Table A1). The majority (66%, *n* = 224) purchased their forage from a producer outside the farm, while the remaining proportion used forage produced on the farm (Table A1). About half (48%, *n* = 163) of the respondents stated that they did not know the forage nutritive contents (Table A1).

More than half of the horses (56%, *n* = 190) were fed concentrates, and the most common type was commercial concentrates (*n* = 118) followed by vegetable oil (*n* = 104) and molassed sugar beet pulp (*n* = 22) and (Table A1). Supplemental feeds were used for 84% (*n* = 285) of the horses in the study and mostly comprised mineral and vitamin feed (Table A1). For horses reported to be fed concentrates, 217 horses were fed concentrates 1–2 times per day and the remaining proportion was fed concentrates more often (Table A1). The presence of FFL was reported to diminish when changing from wrapped forages to hay (58%, *n* = 197), from wrapped forage to pasture (46%, *n* = 156) and from one batch of wrapped forage to another batch (17%, *n* = 58) (Table 4). However, not all horses showed any change in the presence of FFL with feed changes (7%, *n* = 24) and not all horses had been subjected to all feed changes (2%, *n* = 7) (Table 4).

### 3.4. Gastrointestinal Health

**Figure 5:**
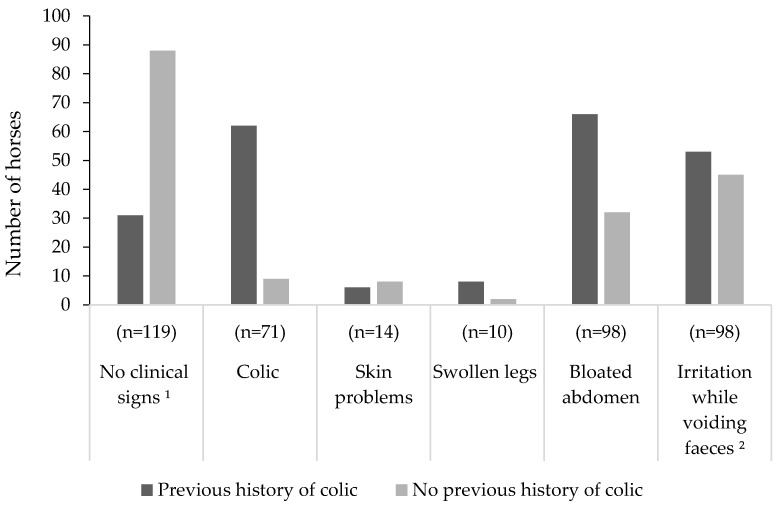
The horse owner reported clinical signs during episodes of free faecal liquid in horses with (*n* = 77) and without (*n* = 256) a previous history of colic. Multiple signs could be selected in the survey, resulting in the numbers of horses for all symptoms exceeding the total number of horses in the study. ^1^ No clinical signs mean no signs other than FFL. ^2^ Including extensive tail swishing and/or trampling with hindlegs.

Eighty-seven percent (*n* = 295) of the horses in the study had not been treated for any gastrointestinal disease within the 3 previous months prior to responding to the survey. Twenty-two percent (*n* = 76) of the horses in the study were reported to have been examined for stomach ulcers, however, only 14 were reported to have been examined with gastroscopy. Of these, 9 were diagnosed with gastric ulcers. Nearly one-quarter of the horses in the study (23%, *n* = 78) were reported to have had a previous history of colic. Therefore, horses were divided into two groups, one with a previous history of colic and one with no previous history of colic, and compared within each type of symptom they were showing during episodes of FFL (Figure 5). For horses reported to show no clinical signs during episodes of FFL (*n* = 119), there was a higher proportion of horses with no previous history of colic (74%, *n* = 88) compared to horses with a previous history of colic (26%, *n* = 31) (*p* < 0.001). For horses reported to show signs of colic during episodes of FFL (*n* = 71) there was a higher proportion of horses with a previous history of colic (87%, *n* = 62) compared to horses with no previous history of colic (13%, *n* = 9) (*p* < 0.001).

For horses reported to show a bloated abdomen (*n* = 98), there was a tendency (*p* = 0.08) for a higher proportion of horses with a previous history of colic (67%, *n* = 66) compared to horses with no previous history of colic (33%, *n* = 32).

### 3.5. Stereotypic Behaviour

Nineteen percent (*n* = 64) of the horses in the study were reported to show stereotypic behaviour. The reported stereotypic behaviours included crib biting (15%, *n* = 8), wind sucking (4%, *n* = 2), weaving (7%, *n* = 4), box walking (11%, *n* = 6), wood chewing (60%, *n* = 33) and tongue rolling (7%, *n* = 4).

## 4. Discussion

### 4.1. Horses

In the present study, the typical horse showing FFL was reported to be of warmblood type, have bay coat colour, be a gelding, on average 12 years old and be used for leisure riding, dressage or show jumping. A similar distribution of horse characteristics has been described within other horse populations in Sweden and Norway [14,15], which indicates that the population in the present study is a reflection of the normal horse population. As a large variety of breeds, ages and disciplines were represented in the population in the present study, it is evident that FFL could appear in almost any type of horse. The proportion of geldings was larger compared to the proportions of mares and stallions in the current study, which is in agreement with the findings in a previous study on FFL [1] where a larger proportion of geldings was found in the case group compared to controls. However, it may be more common to keep geldings as leisure horses [16,17], compared to mares and stallions, and this could explain the higher proportion of geldings in both studies. The reported BCS was in relation to what was expected. The majority of horses were reported to have a BCS of 3 (normal BCS), which is in agreement with previous descriptions of FFL [1,5] where horses were not reported to show weight loss or loss of BCS. It has also been reported that horse owners are commonly underestimating the body condition score of their horses compared to a trained professional scorer [18]. This may indicate that the horses in the present study were not underweight or in lower than normal BCS. In a previous study, horses with the coat colour paint were over-represented (29%, *n* = 12) in a group of horses showing FFL compared to horses in two control groups, which comprised 10% (*n* = 4) and 8% (*n* = 3) paint coloured horses, respectively) [1]. In the current study, the proportion of paint horses was lower than the proportions of the bay, chestnut, grey and black horses. Therefore, an association between the coat colour paint and the presence of FFL cannot be confirmed from the results in the current study. It has been suggested that grey horses may be more prone to show FFL due to the higher risk of melanoma [19], which may cause defecation difficulties, but no such association has been identified in the literature or in this study. Social stress has been suggested to play a role for the presence of FFL, as the majority of FFL-affected horses did not defend their feed against other horses and were judged as low in the hierarchy in a previous study [1]. However, in the same study [1], high ranked horses also showed FFL, indicating that stress from being low in the hierarchy is not a sole explanation for the presence of FFL.

### 4.2. Management

The majority of the horses in this study were kept outside in paddocks for 4–8 h in soil or sand/gravel paddocks and almost half the horses were fed forage on the ground. Feeding forage on the ground could lead to increased ingestion of soil and sand particles causing irritation of the intestinal mucosa, which has been associated with gastrointestinal conditions such as diarrhoea, including voiding of loose and watery faeces [20], and colic [21]. Whether or not this management factor plays a role in the presence of FFL remains to be further elucidated.

### 4.3. Feeding

Despite the fact that about half of the horses were reported to have less loose faeces when feeding was changed from wrapped forages to hay (58%) or to pasture (46%), this was not the case for all horses in the study. In addition, about one-quarter of the horses improved by changing from one batch to another batch (including from primary to regrowth harvest) of wrapped forage. This indicates that the occurrence of FFL cannot be generally attributed to feeding wrapped forages. This finding is supported by results from a previous study in which horses displaying FFL predominantly were fed hay [1]. In addition, FFL has not been reported in controlled feeding studies with healthy horses fed silage, haylage and hay from the same grass sward and harvest [22,23]. As there may be individual variation in the gut microbiota of horses [24,25,26], it is possible that individuals respond differently to the same feed. Further study within this area is highly interesting and may provide more insight into factors contributing to the presence of both FFL and colic.

Although the forage conservation method may influence both chemical and microbial composition in forage [22], forages differ in a number of other factors as well. One important factor is plant maturity at harvest, which greatly influences overall digestibility in the equine GIT and the nutritive value of the forage [27]. Valle et al. [5] reported a horse with FFL to maintain a reduced or absent production of FFL with gradual changes in the nutritional plan to meet the theoretical nutritional requirements of the horse. Changes included reducing energy content in the feed ration by excluding concentrate feeds and decreasing the amount of forage and changing batch of forage in combination with increased exercise. However, simultaneously with the nutritional changes, the horse was treated with sulfasalazine, making it difficult to evaluate the effect of the changes in the feeding. Only half of the horse owners in the current study reported that they knew the nutritive content in their forage (through forage analytical reports), indicating that half of the horses may have been under- or overfed in relation to their nutritional requirements. Whether this is a factor of importance for the presence of FFL is currently not known.

The composition of the total feed ration and the ratio between forage and concentrate may be of importance for the physical appearance of faeces, as two-phase separation (liquid and solid phase) of both digesta and faeces has been reported in horses fed hay ad libitum with inclusion of grains (4.55 kg every 12 h) but not when fed only the same hay ad libitum [28]. In horses fed hay only, a clear separation between solid and liquid phases was present in the contents of the right dorsal colon (RDC), but faecal balls were well-formed and with no separation in liquid and solid phases. No or minimal gas bubbles were present in RDC content when horses were fed hay [28]. When horses were fed hay with the inclusion of grains, RDC contents were more homogenous and foamy, with less separation of phases, and the liquid phase was more viscous than in horses fed hay only. Faeces of horses fed hay and grains were, however, less formed and had a clear separation, where the liquid phase had noticeable gas bubbles and was more viscous compared to faeces of horses fed hay only [28]. One explanation for this result could be differences in the hydrophilic properties of ingesta components, which may differ when horses were fed hay only or hay with grain inclusion. The hydrophilic properties of the ingesta have been suggested as a cause of osmotic diarrhoea [29,30]. In the current study, horses were fed much smaller proportions of concentrates compared to what was described by Lopes et al. [28]. However, smaller amounts of concentrates fed daily (2.5–5 kg) have been reported to increase the risk of colic [31,32,33] and may affect the ingesta and its transit as well. As horses displaying FFL seldom show clinical signs of disease, FFL may be a type of osmotic diarrhoea. Further insights in causes of FFL may be provided by investigations of forage and concentrate proportions in the total feed ration of horses with FFL.

### 4.4. Gastrointestinal Health

In previous studies, no symptoms of disease, such as fever or weight loss or loss of body condition has been described in horses with FFL [1,5]. Nine horses were reported to have been diagnosed with gastric ulcers, but overall very few horses (*n* = 14) had been examined for gastric ulcers with gastroscopy. From the results of the current study, it cannot be ruled out that gastric ulcers may be associated with the presence of FFL, even though the incidence of gastric ulcers was lower than what has been described in other studies of gastric ulcers in leisure horses (17% to 58%, [34,35,36]).

In the present study, almost one-quarter of the horses were reported to have had a previous history of colic. A colic incidence between 3.5% and 10.6% has been reported for general horse populations [10,32,37,38] and of 4.8% within a German population of horses showing FFL [3]. This indicates that the incidence of colic was higher for Swedish and Norwegian horses showing FFL. In addition, in the present study, 55% of the horses did not show any clinical signs other than faecal liquid during FFL episodes, whereas the remaining proportion of horses were reported to have one or a combination of several clinical signs including e.g., colic symptoms. The latter proportion also had a higher number of horses with a previous history of colic. This indicates that causes of FFL could differ among different horses, or that FFL is a generic symptom from several different conditions of a similar nature. The number of clinical signs during an FFL episode could also depend on the severity of the condition, which could not be assessed in this study. Further studies of FFL should preferably include detailed descriptions of duration, intensity and severity of FFL episodes as well as previous disturbances in the GIT of FFL-affected horses. It is possible that the hindgut microbiota of FFL-horses is responsible for the clinical signs. Transplantation of faecal microflora in affected horses has been reported to decrease the severity of FFL in a controlled study [4]. However, in the same study [4], horses were also treated with omeprazole and psyllium seeds in addition to faecal transplant, making it difficult to evaluate the effects.

### 4.5. Stereotypic Behaviour

The reported incidence of stereotypic behaviour among FFL horses in the study was 19% when wood chewing was included. Tree-wood chewing may not always be a stereotypy but could be related to low-fibre diets [39], and when excluded, the incidence of stereotypies was approximately 8%, which is comparable to the previously reported incidence of 7.1–12.3% [40,41,42,43,44] in other horse populations. Factors reported to be associated with an increased risk of stereotypic behaviour in previous studies include low levels of social interactions with other horses [44,45,46], low forage availability [45,46] and low number of horses kept in the same and/or adjacent paddocks [45]. The majority of horses in the current study were kept in individual boxes when stabled and fed forage in predetermined portions during the day, which may have contributed to the incidence of stereotypic behaviour among horses in this study.

### 4.6. Survey Response and Limitations of the Study

The advantages of performing online surveys are the ease and low cost of data collection, the automation in data input and handling, which reduce errors, and the flexibility in survey design to make it easier for participants to respond to the questionnaire. The disadvantage includes the absence of an interviewer, and that data are reported by the horse owner, which could result in misinterpretations among respondents for some of the questions. Some of the variables have previously been shown to be difficult to estimate correctly for horse-owners, such as BCS [18]. It should be recognized that all conclusions drawn from this study were based on the perceptions of the respondents, which may vary in their knowledge of equine feeding and management.

In order to control that the respondents of the survey were within the intended group, control questions based on the inclusion criteria, such as the age of the horse, and if the horse had problems with FFL when fed wrapped forages, were asked. This resulted in the elimination of 441 responses to the present study, indicating that such controls could be of high importance to enhance the quality of data input in online surveys.

## 5. Conclusions

There was a large variety of horse characteristics, including breed type, age and coat colour, in horses with FFL. Many, but not all horses in this study were reported to show less separation of solid and liquid phases in their faeces when changing from wrapped forages to hay, pasture or another batch of wrapped forage. Horses with FFL were also reported to have a higher incidence of a previous history of colic compared to reports from other horse populations. Further research on FFL in horses is of interest and should include details on feeding (such as forage nutritive values, feed ration composition), and gastrointestinal tract health and function (such as the presence of stomach ulcers, colic, gastrointestinal tract response to different feedstuffs), as well as detailed descriptions of severity and duration of FFL episodes.

## Figures and Tables

**Table 1:** Characteristics of horses showing free faecal liquid (*n* = 339).

Item	No. of Horses	% of Horses
Country (stabled in)		
Sweden	191	56
Norway	148	44
Gender		
Mare	134	40
Gelding	194	57
Stallion	11	3
Coat colour		
Bay	123	37
Chestnut	64	19
Grey	47	14
Black	27	8
Paint	24	7
Palomino/Isabelline	21	6
Cremello	19	6
Other (Leopard pattern/buckskin)	14	4
Body condition score ^1^		
<3	75	22
3	188	55
>3	76	22
Training intensity		
Low	215	63
Medium	63	19
High	23	7
Breaking in	23	7
No training ^2^	15	4

^1^ According to the scale of Carroll and Huntington, 1988. ^2^ No training includes horses kept as pets or for company.

**Table 2:** Description of the management of horses showing free faecal liquid (*n* = 339, if not otherwise mentioned. Deviances in N were due to missing responses for that particular question).

Item	No. of Horses	% of Horses
Housing system (*n* = 337)		
Individual box	271	79
Loose housing system	64	19
Group housing	2	1
Bedding (*n* = 336)		
Straw	67	20
Shavings	57	17
Combination of straw and shavings	125	37
Sawdust	40	12
Wood pellets	26	8
Straw pellets	11	3
Other (paper, mix of sawdust and peat, rubber mat, raw sawdust)	10	3
Time spent per day in paddock during winter (*n* = 245)		
<4 h	5	2
4–7 h	67	20
8–12 h	163	48
>12 h	10	30
Paddock ground (*n* = 332)		
Grass (old grass during winter)	94	28
Sand/Gravel	79	24
Soil	133	40
Other	26	8
Annual time spent on pasture		
<4 weeks	5	2
4–8 weeks	67	20
9–12 weeks	163	48
>12 weeks	100	30
No pasture	4	1
Anthelmintic routines		
Regularly dewormed ≥ 1 times per year	122	36
Dewormed due to high ^1^ egg counts ≥ 1 times per year	154	45
Dewormed due to high ^1^ egg counts < 1 times per year	34	10
Dewormed if considered necessary	25	7
Not dewormed	4	1

^1^ According to national guidelines (www.sva.se).

**Table 3:** Daily amounts of different feedstuffs (kg per 100 kg bodyweight (BW) per day) and proportion (%) of roughage and concentrate in the diet offered to horses showing free faecal liquid (*n* = 339).

Item	No. of Horses	Min	Q1	Q2	Q3	Max	Mean	SD
Roughage and concentrate feeding, Kg/100 kg BW/d ^1^								
Grass hay	165	0.1	0.2	0.2	0.3	0.5	0.2	0.06
Grass haylage	251	0.2	1.7	2.0	2.3	6.0	2.0	0.67
Grass silage	4	1.2	1.2	2.9	3.8	4.7	2.9	1.73
Straw	14	0.1	0.2	0.3	0.6	2.3	0.4	0.45
Lucerne ^2^	10	0.01	0.1	0.1	0.2	0.7	0.1	0.13
Total amount of roughage	217	0.3	1.5	2.0	3.1	4.8	1.8	2.17
Total amount of concentrate	190	0.01	0.1	0.2	0.3	1.0	0.2	0.18
Roughage proportion of total feed ration (%) ^3^	249	20	90	100	100	100	90	0.14
Concentrate proportion of total feed ration (%) ^3,4^	107	0	1	5	10	80	7	0.14
Mineral supplementation, g/100 kg BW	218	0.1	6.0	10.8	17.8	83.3	13.5	11.43

^1^ Horses reported to have ad libitum access to roughage, forage or having straw as bedding material were not included. ^2^ Horses reported to have access to roughage ad libitum without concentrates in the diet were included. ^3^ Horses reported to have access to roughage ad libitum without concentrates in the diet were included. ^4^ Horses reported not to be fed concentrate were excluded. Min = Minimum value. Q1–Q3: First-, second- (=median) and third quartile. Max = Maximum value. SD = Standard deviation.

**Table 4:** Changes in the presence of free faecal liquid in the horses in the study (*n* = 339) with diet changes as reported by respondents. “Less loose” refer to the absence and/or reduced amount of liquid phase in faeces compared to before the feed change, as reported by respondents. Not all respondents had tried all response alternatives.

Item	No. of Horses	% of Horses
Faecal appearance less loose when changing from wrapped forage to hay	198	58
Faecal appearance less loose when changing from wrapped forage to pasture	157	46
Faecal appearance less loose when changing to another batch of wrapped forage	56	17
No change in faecal appearance with any change in feeding	24	7
Faecal appearance more loose in association to changing feeds	20	6
Faecal appearance less loose when changing from primary to regrowth harvest ^1^	16	5
Faecal appearance less loose when using feed additives ^2^	8	4
Have not tried any change in feeding	5	2

^1^ Wrapped forages. ^2^ Feed additives reported included yeast, linseed, psyllium seed, thiamine and various types of commercial pro- and prebiotics.

## Supplementary Materials

The following are available online at https://www.mdpi.com/2076-2615/10/1/76/s1, Table S1, The survey distributed to Swedish and Norwegian horse owners having horses showing FFL when fed wrapped forages. Modified (Translated from Swedish and Norwegian language) for the purpose of publication. Bulleted points indicate the response of the questions and different response alternatives are comma-separated. Space was provided for alternative answers where necessary.

## Abbreviations

The following abbreviations are used in this manuscript:

BCS	Body Condition Score
DM	Dry matter
FFL	Free faecal liquid
FMT	Faecal microbiota transplantation
GIT	Gastrointestinal tract
RDC	Right dorsal colon

## Appendix A

Horse owner responses to the survey for variables not presented in tables or figures in the main text. The number (*n*) and proportion (%) of horses presented are presented in Table A1.

**Table A1:** Extended information on management factors and feeding strategies for horses with free faecal liquid (*n* = 339), as reported by respondents.

Variables	Total Number of Horses	% of All Horses
Horses showing FFL when fed wrapped forages	339	100
Region of stable		
Southern	126	37
Central	81	24
North	39	12
Western ^1^	23	7
Eastern ^1^	70	21
Horse imported		
No	253	75
Do not know	6	2
Yes	80	24
Ability of horse to keep desired BCS		
Easy keeper	97	29
Normal	187	55
Hard keeper	55	16
Type of water source in stable/loose housing system		
Frostless waterer	49	14
Frostless tub	43	13
Waterer	31	9
Tub	31	9
Bucket	78	23
Natural water source	4	1
Combination of bucket and waterer	103	30
Type of water source in paddock during winter		
Frostless waterer	34	10
Frostless tub	80	24
Waterer	4	1
Tub	124	37
Bucket	37	11
Natural water source	5	1
Other (Combination of tub and bucket, bucket and waterer)	55	16
Type of pasture		
Pasture on arable land	106	31
Natural pasture	135	40
Forest	6	2
No pasture	30	9
Other (Combination of different pasture types)	61	18
Type of water source on pasture		
Frostless waterer	45	13
Frostless tub	12	4
Waterer	10	3
Tub	165	49
Bucket	8	2
Natural water source	33	10
Other (Combination of bucket and tub)	66	19
Access to saltlick while on pasture		
Yes	220	65
No	119	35
Saltlick in stable/loose housing system		
Yes	296	87
No	43	13
Time from last deworming		
Not dewormed	11	3
0–3 months ago	75	22
4–6 months ago	126	37
7–12 months ago	68	20
>1 year ago	59	17
Origin of the forage		
Bought	226	67
Produced on farm, but not by the owner	42	12
Produced on farm by the owner	69	20
Other	1	0
Forages analysis		
Yes	144	42
No	163	48
Do not know	37	9
Number of feedings of forage per day		
1 time	1	0
2 times	14	4
3 times	122	36
4 times	108	32
>4 times	33	10
Free access	89	26
Storage of forage		
Indoors	223	66
Outdoors	114	34
Outdoors, covered	65	19
Outdoors, uncovered	49	14
Hay indoors, wrapped forages outdoors uncovered	1	0
Hay indoors, wrapped forages outdoors covered	1	0
Maximum time between two feedings of roughage		
0–2 h	2	1
2–4 h	35	10
4–8 h	115	34
8–12 h	101	30
>12 h	8	2
Don’t know	78	24
Feeding strategy for roughage in paddock ^2^		
Forage not fed in the paddock	27	8
On the ground	113	33
In the feeding rack	79	23
In a haynet	19	6
In a tub or similar	62	18
Other (combination of ground and feeding rack/haynet)	39	12
Type of concentrate fed ^2^		
Grains	16	5
Molassed sugar beet pulp	22	6
Linseed/Linseed cake	4	1
Soybean meal	14	4
Potato protein	33	10
Wheat bran	3	1
Vegetable oil	104	31
No concentrate fed	149	44
Commercial concentrate	118	63
Number of concentrate feedings per day		
0 times	149	44
1 time	116	34
2 times	107	32
3 times	49	14
4 times	7	2
>4 times	1	0
Type of supplemental feeds fed ^2^		
Mineral feeds	237	70
Multivitamins	58	17
B-vitamins (and Biotin)	10	3
Selenium and Vitamin E	9	3
Garlic	13	4
Herbs	8	2
Other (Yeast, magnesium)	93	27
Not fed supplemental feeds	51	16
Storage of concentrates		
In covered/closed containers indoors	255	66
In uncovered/open containers indoors	11	3
In paper bags/original package indoors	18	5
No concentrate	51	15
Other	4	1
Previous treatment of other gastro-intestinal diseases		
No	296	87
Don’t know	11	3
Yes	32	9

^1^ Only for Norwegian respondents. ^2^ Multiple choice question.
