# Supplementary material for: Differential Defecation of Solid and Liquid Phases in Horses—A Descriptive Survey
Source: Animals (Basel). 2020 Jan 1;10(1):76. doi: 10.3390/ani10010076 (PMC7023164; doi:10.3390/ani10010076)
Supplement: Supplementary file 1 [file animals-10-00076-s001.pdf]

Table S1. The survey distributed to Swedish and Norwegian horse owners having horses showing FFL when fed wrapped forages. Modified (Translated from Swedish and Norwegian language) for the purpose of publication. Bulleted points indicate the response of the questions and different response alternatives are comma-separated. Space was provided for alternative answers where necessary.

1. In which region is your horse stabled?

   - Northern Sweden/Norway, Central Sweden/Norway, Southern Sweden/Norway

2. How old is your horse? (Years): ______

3. Which breed is your horse? *If crossbred, enter the breeds you know*

   - Arabian, Angoloarabian, Thoroughbred, Swedish warm-blood (SWB), Standardbred, Cold-blood trotter, North Swedish draught-horse, Ardenneais, Gotland pony, Shetland pony, Connemara pony, New Forest pony, Welsh pony, Welsh Cob, Friesian horse, Haflinger, Quarter horse, Paint horse, Appaloosa, Tinker horse, Clydesdale, Shire horse, Icelandic horse, P.R. E (Pura Raza Española, Andalusian), Lusitano, Riding pony, Crossbred: ____________

4. Which gender is your horse?

   - Mare, Gelding, Stallion

5. Is your horse born and bred in Sweden/Norway?

   - Yes, Don't know, No; imported from: _____________

6. What is the colour of your horse? *Choose the colour closest to the colour of your horse.*

   - Grey, Bay, Black, Chestnut, Paint, Palomino/Isabelline, Leopard pattern, Buckskin, Cremello, Other: ________________________________

7. What is the withers height of your horse? *Type the answer in cm.*_________________________

8. What is the weight of your horse? *Type the answer in kg. Help: Approximate weight for different breeds: Shetland pony 100-200 kg, Gotland pony 150–250 kg, Icelandic horse 250–400 kg, Arabian horse 350–500 kg, Thoroughbred 400–600 kg, Standardbred 400–600 kg, Swedish warmblood (SWB) 450–700 kg, Ardenneais 700–900 kg.* ____

9. My horse is:

   - An easy keeper (needs less feed than the average horse to maintain its body condition), A hard keeper (needs more feed than the average horse to maintain its body condition), A normal keeper.

10. The body condition score (BCS) of my horse is at the moment:

    - 0, 1, 2, 3, 4, 5

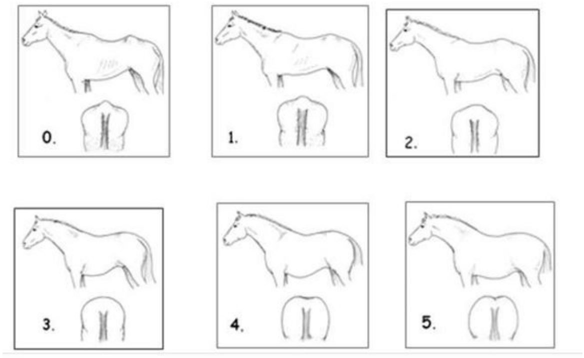

11. I think my horse is (*Multiple responses possible)*:

- Calm, Nervous, Curious, Introvert, Happy, Tense, Alert, Lazy, Hardworking, Unwilling, Stressed, Uninterested, Active, Passive, Irritable, Angry

12. Which disciplines do you perform with your horse? *(Multiple responses are possible)*

- Dressage, Showjumping, Cross country, Leisure riding, Riding school, Breeding show, Breeding, Western, Working equitation, Endurance, Racing, Trotting, Pet/Company, Academic art of riding, Jousting/Mounted archery, Natural horsemanship, Liberty, Breaking in, Other:_______

13. Which training intensity is consistent with your horse's training? *Choose one option*

- Very low (maximum 30 min/day, 1–3 days/week, mainly in walk), Low (e.g., leisure riding, 30–60 min per day, 4–7 days/week, all gaits), Medium (e.g., riding school, more intense leisure riding, all gaits), High (e.g., cross country, high-level show jumping, all gaits), Very high (Trotting and racing, high-level cross country training, endurance), Breaking in, Others:_______

14. My horse is kept as follows during winter:

- Individual box at night, paddock with other horses during daytime, Individual box at night, alone in paddock during daytime, Individual tied up stall during night, paddock with other horses during daytime, Individual tied up stall during night, alone in paddock during daytime, Group housing during night, paddock with other horses during daytime, Group housing during night, alone in paddock during daytime, Loose housing system with other horses, Kept alone in a loose housing system, Other: ________________________________

15. How long does your horse spend outside in a paddock during wintertime? *Type your answer in number of hours per day.*____________

16. What type of paddock is your horse kept in during wintertime?

- Grass paddock with grass all year round (old grass during winter), Sand/Gravel, Soil/Clay, Other type of paddock: ____________

17. Which bedding material(s) do you use in your horsebox/stall/loose housing system? *(Multiple responses are possible)*

- Straw, Shavings, Sawdust, Wood, Paper, Mix of sawdust and peat, Rubber mat, Raw sawdust, Straw pellets, Sawdust pellets, Other:_________________

18. My horse has access to water in the stable/loose housing system in the following way during winter;

- Frostless waterer, Frostless tub, Waterer, Tub, Bucket, Natural water sources, Other:___________

19. My horse has access to water in the paddock in the following way during winter;

- Frostless waterer, Frostless tub, Waterer, Tub, Bucket, Natural water sources, Other: ___________

20. Does the horse have access to a saltlick in the stable/loose housing system?

- Yes, No, Yes; and also gets extra salt in feed, Yes; and also gets extra salt in special water buckets, No; gets extra salt in feed instead, No; gets extra salt in special water buckets, Other_________________

21. Is your horse kept at pasture during summer?
(Meaning that the horse covers all or part of its nutritional requirements from grass)

- Yes; less than 4 weeks, Yes; 4–8 weeks, Yes; 8–12 weeks, Yes; longer than 12 weeks, No, Other: _______

22. Which type of pasture is your horse kept at during summer?

- Pasture established on cropland, Natural pasture, Forest, No pasture, Other type of pasture: ________

23. Does your horse have access to a saltlick while on pasture?

- Yes, No, Yes; and also gets extra salt in feed, Yes; and also gets extra salt in special water buckets, No; gets extra salt in feed instead, No; gets extra salt in special water buckets, My horse is not let out on pasture, Other: ________________________________

24. My horse has access to water at the pasture in the following ways during summer;

- Frostless waterer, Frostless tub, Waterer, Tub, Bucket, Natural water sources, Other: _____________

25. Which of the following best describes your deworming routines?

- The horse is dewormed regularly at least once a year, The horse is dewormed when decided by the owner, The horse is dewormed when needed based on faecal egg count at least once a year, The horse is dewormed when needed based on a faecal analysis less than once a year, The horse is not dewormed due to parasite free pastures, The horse is not dewormed due to parasite free pastures as it has not been grazed by horses/donkeys for several years, The horse is not dewormed, Other: ______________________________

26. When was your horse last dewormed?

- I have never dewormed my horse, 0–3 months ago, 3–6 months ago, 6–12 months ago, >1 years ago, Other: _______________

27. Which roughage(s) is your horse fed at the moment? *Choose one or more options.*

- Small bale hay, Big bale hay, Loose hay, Big bale grass haylage (at least 50% DM), Small bale grass haylage (at least 50% DM), Big bale grass silage (under 50% DM), Small bale grass silage (under 50% DM), Straw, Lucerne, (pelleted) Lucerne (chopped), Other: ________________

28. Is the forage bought or produced on the farm?

- Bought, Produced on farm (but not by the owner), Produced on farm, by the owner, Other: ________

29. Is the forage analysed for its nutritive contents?

- Yes, No, Don't know______________________________

30. What is the nutritional content of the forage? Please fill in the values per kg dry matter for the forage that you use at the moment.

- Dry matter (%), Energy (MJ/kg DM), Digestible crude protein (g/kg DM), Ca (g/kg DM), P (g/kg DM), Mg (g/kg DM):_____________________________________

31. Do you feed your horse any concentrate (s)?

- Yes, No

32. Which type of concentrate(s) do you feed your horse? *Choose one or more options.*

- Oats, Barley, Molassed sugar beet pulp, Linseed/linseed cake, Soybean meal, Potato protein, Wheat bran, Vegetable oil, No concentrate(s), Other (write brand and type): _______________

33. Do your horse get any supplement feeds? (E.g. mineral feeds, vitamin feeds, herb supplements etc.)

- Yes, No

34. What type of supplemental feeds do you give your horse?

- Mineral feeds, Multivitamin feeds, B-Vitamin feeds, Selenium + E-vitamin additive, Garlic, Herbs, Do not feed any concentrate, Other (specify brand and type): ______________________________

35. Which amounts (gram or kg) of feed is your horse fed per day? Write 0 in the box if your horse is not fed that type of feed. If your horse is fed several types of feeds in the same category, write type of feed and specific amount for each type of feed e.g., "3 kg hay and 5 kg grass haylage".

- Forage (including hay, grass haylage and glass silage) (kg/day), Concentrate (kg/day), Straw (kg/day), Lucerne (kg/day) Additional feeds (g/day)

36. How many times per day is your horse fed roughage?

- 0 times, 1 times, 2 times, 3 times, 4 times, >4 times, Free access

37. How many times per day is your horse fed concentrate?

- 0 times, 1 times, 2 times, 3 times, 4 times, >4 times, Free access

38. How many hours is it at the most between two feedings of roughage?

- 0–2 h, 2–4 h, 4–8 h, 8–12 h, >12 h, Free access, Don't know

39. How is the forage fed in the paddock?

- Forage is not fed in the paddock, On the ground, In a feeding rack, In a haynet, In a tub or similar, Other way: ________________________________

40. How do you store your forage? (If you feed your horse wrapped forage the question concerns opened bales).

- Indoors (stall, barn or similar), Outdoors (under roof), Outdoors (no roof), Other: __________________

41. How do you store your concentrate feeds?

- In covered/closed container indoors, Uncovered/open container indoors, In paperbags/original package indoors, Do not feed concentrate, Other________________________________

42. Has your horse showed loose faeces when fed wrapped forages?

- Yes—generally loose faeces which looks like "cow pat", Yes, solid faecal balls but also free faecal liquid—Yes, diarrhoea without solid faecal balls, No, Other: ________________________________

43. If your horse has showed loose faeces when fed wrapped forages, has it become better or good when:

- Changing from wrapped forages to hay, Changing from one batch of grass haylage to another batch of grass haylage, Change from primary harvest to regrowth harvest, Change from wrapped forages to pasture, No improvement with any tried change, Worsened condition with any tried change, My horse have never had any problems with loose faeces when fed wrapped forages, Other: ___________

44. If your horse has shown loose faeces when fed wrapped forages, have other horses in your stable fed the same forage also shown loose faeces?

- No; only my horse, Yes—several horses, My horse have never had any problem with loose faeces when fed wrapped forages, Don't know, If "yes", write the number of horses (e.g., 2 out of 10):____________

45. Has your horse shown loose faeces when fed hay?

- Yes—generally loose faeces who looks like "cow pat", Yes—solid faecal balls but also free faecal liquid, Yes—diarrhoea without solid faecal balls, No, Other: ________________________________

46. Has your horse showed any of the following signs during an episode of loose faeces when fed wrapped forages and/or hay? *Choose one or more options*

- My horse have never showed loose faeces when fed wrapped forages or hay, Colic, Skin problems (e.g., nodules and urticaria, but not summer eczema), Swollen legs not caused by training or injury, Bloated abdomen, Irritation while voiding faeces (swishing tail and/or trampling with hindlegs), None of the options, Other: ____________________

47. Does your horse have a history of previous colic episodes?

- Yes, No, Don't know

48. Has your horse been examined and diagnosed with gastric ulcers by a veterinarian?

- Yes—my horse has been examined by a veterinarian and has been diagnosed with gastric ulcers, Yes—my horse has been examined by a veterinarian but has not been diagnosed with gastric ulcers, No—not examined, Don't know

49. Has your horse been treated by a veterinarian for any other diseases/conditions in the gastro-intestinal tract?

- No, Don't know, Yes—my horse has been treated for:______________________

50. Does your horse show any of following behaviour?

- Crib biting, Wind sucking, Weaving, Box walking (walk around in the box in a repeated pattern), Self-biting (bites itself on the sides/flanks), Wood chewing (e.g., stable interior, fence, but not trees and bushes), Tongue rolling ("chewing on the tongue" in a repeated pattern, e.g., before feeding), My horse do not show any of the behaviours listed, Other: ________________________________
